# Can Recreational Soccer Improve Physical Literacy Dimensions Among Adolescents with High Cardiometabolic Risk?

**DOI:** 10.3390/sports13120423

**Published:** 2025-12-02

**Authors:** Gustavo Pavez-Adasme, Juan Párraga-Montilla, Cristián Martínez-Salazar, Marcelo Castillo-Retamal, Pedro Latorre-Román, Nicolás Gómez-Álvarez

**Affiliations:** 1 Centro Regional de Estudios Avanzados en Estilos de Vida Activos y Saludable (CREA-EVAS), Universidad Adventista de Chile, Chillán 3780000, Chile; gustavopavez@unach.cl; 2 Departamento de Didáctica de la Expresión Musical, Plástica y Corporal, Universidad de Jaén, 23001 Jaén, Spain; jparraga@ujaen.es (J.P.-M.); platorre@ujaen.es (P.L.-R.); 3 Centro de Investigación en Alfabetización Motriz, Universidad de La Frontera, Temuco 4780000, Chile; 4 Departamento de Educación Física, Deportes y Recreación, Universidad de La Frontera, Temuco 4780000, Chile; cristian.martinez.s@ufrontera.cl; 5 Departamento de Ciencias de la Actividad Física, Universidad Católica del Maule, Talca 3460000, Chile

**Keywords:** physical literacy, small-sided soccer, overweight, obesity, cardiometabolic risk

## Abstract

This study aimed to analyze whether recreational soccer, through small-sided and traditional soccer formats, can promote improvements in different dimensions of physical literacy among adolescents with high cardiometabolic risk. Methodology. A randomized controlled trial was conducted with two experimental conditions (small-sided soccer games and traditional soccer) and one control condition. A total of 51 male adolescents aged 11–15 years with high cardiometabolic risk participated in the study. A model including three dimensions of physical literacy was established: physical, psychological, and cognitive dimensions. Data were standardized using z-scores to allow variable weighting within the model, and effect sizes and ANCOVA were used for inter- and intragroup comparisons. Results. The overall effect on physical literacy was small (ES: 0.31). The small-sided soccer group showed better results in the physical and psychological dimensions. The per-protocol analysis showed small effects in the high-adherence groups (ES = 0.43 and 0.38) and significant within-group differences; however, the post hoc analyses only revealed trends when compared with the low-adherence groups. Conclusions. Recreational soccer, whether in SSG or TSG formats, was insufficient to elicit significant improvements in physical literacy among adolescents with high cardiometabolic risk. Participants with higher adherence showed better outcomes, with SSG showing a clear tendency toward greater improvements in the physical and psychological domains.

## 1. Introduction

Pediatric obesity is recognized as one of the main global public health problems [1,2], given its close association with multiple metabolic disorders such as arterial hypertension, type 2 diabetes, metabolic syndrome, and increased cardiovascular mortality—conditions that tend to persist into adulthood [3]. Chile ranks second worldwide in the prevalence of obesity among adults [4], while in the pediatric population, it is positioned as the fifth country with the greatest increase in childhood obesity [5], with a current prevalence of overweight and obesity of 50.9% among children and adolescents, implying an increased cardiometabolic risk from early stages [6]. Although this problem is multifactorial [7], physical activity (PA) and exercise are key strategies to counteract these risks [8].

Although physical exercise has been shown to be effective in improving cardiometabolic health, its effectiveness in real-world settings is often debated due to problems with adherence and low sustainability of long-term benefits [9]. In this context, the recent development of the concept of physical literacy (PL) provides an integrative theoretical framework that can enhance PA-based intervention programs by recognizing the importance of motivation, confidence, knowledge, and motor competence needed to maintain an active and healthy lifestyle throughout life [10]. This construct, which has been widely studied [11,12], promotes adherence to and enjoyment of motor activities across the lifespan [13,14]. Among adolescents with high cardiometabolic risk, PA becomes particularly relevant, as it addresses not only physiological parameters but also psychological, cognitive, and social interaction barriers, which can be strengthened through participation in sports activities—thereby improving engagement and the sustainability of interventions [15].

Soccer is one of the most popular sports worldwide, characterized as a highly social and enjoyable activity [16] and a promising strategy to enhance health [17,18]. Likewise, small-sided soccer games (SSG)—a modified version of soccer with fewer players, smaller spaces, and adapted rules—have gained popularity due to their effectiveness and versatility in both sport practice [19] and teaching [20]. Evidence from youth populations highlights their ability to promote physical fitness in children and adolescents [21,22], improve cardiometabolic health [23,24,25], enhance agility [26,27], and develop decision-making under pressure [28]. Moreover, its playful and interactive nature increases enjoyment [29] and intrinsic motivation [30], fostering social well-being [31] and inclusive interactions that facilitate its implementation in both school and community settings [32].

The integrative potential of SSG as an intervention strategy is capable of generating benefits across multiple dimensions—physical, psychological, social, and cognitive—as proposed by the PL framework, which justifies the interest in analyzing its effectiveness in promoting PL. However, to the best of our knowledge, no studies have analyzed these benefits from a truly multidimensional perspective, which represents an operationalization challenge already identified within the PL framework [33]. This need is particularly relevant in clinical or at-risk populations [34], such as adolescents with cardiometabolic risk factors, in line with the inclusive perspective that underpins this concept [35].

Therefore, this study aimed to analyze whether recreational soccer, through small-sided and traditional soccer formats, can promote improvements in the different dimensions of PL in adolescents with high cardiometabolic risk. It was hypothesized that the small-sided soccer intervention would be more effective in promoting the development of PL than the traditional soccer format among adolescents with high cardiometabolic risk.

## 2. Materials and Methods

This study was a randomized controlled trial that included two experimental conditions and one control condition. The study was registered at ClinicalTrials.gov (ID: NCT06377137) and adhered to the CONSORT guidelines for reporting randomized controlled trials.

### 2.1. Participants

Participants were 51 male adolescents with abdominal obesity, with a mean age of 12.66 ± 1.20 years, height of 162.55 ± 9.36 cm, and a mean body weight of 76.23 ± 12.06 kg. Abdominal obesity was defined as a waist circumference (WC) ≥ 90th percentile [36] or waist-to-height ratio (WHtR) ≥ 0.50 [37].

The eligibility criteria were as follows: inclusion criteria: (i) males aged 11–15 years at the start of the intervention; (ii) abdominal obesity defined as WC ≥ 90th percentile [36] or WHtR ≥ 0.50 [37]; and (iii) availability to participate regardless of group assignment. The exclusion criteria were as follows: (i) health conditions incompatible with vigorous exercise (e.g., cardiovascular disease, orthopedic or neurological limitations); (ii) significant musculoskeletal injuries within the previous six months; and (iii) participation in structured weight-loss or exercise programs outside of regular physical education classes.

The protocol was approved by the Ethics Committees of the Faculty of Sport, University of Porto (CEFADE 08-21) and Universidad Adventista de Chile (No. 2023-03), and was conducted in accordance with the principles of the Declaration of Helsinki of the 75th General Assembly of the World Medical Association [38]. Recruitment took place between March and April 2023 at four schools in Chillán, Chile. All participants and their legal guardians provided signed assent and informed consent forms.

### 2.2. Materials and Tests

#### 2.2.1. Physical Literacy Measures

To assess the components of physical literacy (PL), the Canadian model was used as a reference, integrating the physical, cognitive, psychological (affective), and behavioral dimensions [39]. Specifically, the second version of the Canadian Assessment of Physical Literacy (CAPL-2) [40] was employed, which includes indicators of physical activity (behavioral dimension), muscular strength and cardiorespiratory endurance (physical dimension), motivation and confidence (psychological dimension), and knowledge and understanding (cognitive dimension) [41].

A PL model was established based on z-scores to standardize the data and weight the variables within the model. The weighting of the dimensions was established in a 50:40:10 ratio for the physical–behavioral, psychological, and cognitive dimensions, respectively, consistent with the proportions used in the CAPL-2 manual [40]. The test applied is detailed below.

#### 2.2.2. Cardiorespiratory Endurance

Cardiorespiratory fitness was assessed using the six-minute walk test (6MWT), following the recommendations of the ATS Committee on Proficiency Standards for Clinical Pulmonary Function Laboratories [42] and previously validated in Chilean adolescents [43]. Maximal oxygen consumption (VO_2_max) was estimated using the equation proposed by Jalili et al. [44]:

VO_2_max (ml·kg^−1^·min^−1^) = 12.701 + (0.06 × distance walked in 6 min [m]) − (0.732 × body mass index [kg/m^2^])

#### 2.2.3. Moderate-to-Vigorous Physical Activity (MVPA)

MVPA was quantified objectively using accelerometry. The participants were instructed to wear an accelerometer on their left wrist for seven consecutive days, including the weekend [45,46]. Actigraph^®^ wGT3X devices (Actigraph L.L.C, Pensacola, FL, USA), were used, configured at a sampling frequency of 100 Hz and a 15 s epoch, following recommendations for this population [47,48]. For analysis, recordings were considered valid if they included at least four complete days (≥8 h per day), with at least one weekend day, according to Trost et al. [49]. Device initialization and data download were performed using ActiLife software (Actigraph, v.6, Pensacola, FL, USA), and subsequent data processing was conducted using the GGIR package v.3.3-2 (R Project for Statistical Computing), with cut-off points specific to pediatric populations [47].

#### 2.2.4. Strength Variables

Lower-body strength was assessed using the horizontal jump (HJ) test, recording the best of three attempts [50]. Upper-body strength was measured using a JAMAR^®^ Plus dynamometer (Chicago, IL, USA), considering the sum of the highest values from each hand as handgrip strength (HGS). In addition, adjusted indicators were calculated: HGS relative to body weight (HGS/relative) and HGS adjusted for BMI to obtain the muscular quality index (MQI) [51,52]. Finally, the General Strength Index (GSI) was calculated following the proposal of Pacheco-Herrera et al. [53], corresponding to the mean of the z-scores for maximal handgrip strength in both hands and the maximal horizontal jump.

$$\mathrm{IGF}=\frac{{HGS}_{MÁX\;ZSCORE}+{SH}_{MÁX\;ZSCORE}}{2}$$

HGS max = z-score of maximal handgrip strength of both hands.

HJ max = z-score of maximal horizontal jump.

### 2.3. Psychological Dimension

#### 2.3.1. Motivation and Confidence

Motivation and confidence were assessed using the corresponding section of the Canadian Assessment of Physical Literacy—second version (CAPL-2) [40], validated in Spanish by Pastor-Cisneros et al. [54], which showed high internal consistency (α = 0.730–0.987). This dimension includes two variables: Adequacy and Predilection (α = 0.918), measured with the “What is more like me?” questionnaire, which consists of six items with response options “very true for me” or “not very true for me,” and Intrinsic Motivation (α = 0.905), evaluated according to the CAPL-2 manual guidelines.

#### 2.3.2. Enjoyment

The level of enjoyment of physical activity was assessed using the Physical Activity Enjoyment Scale (PACES) [55], in its Spanish-translated and validated version by Moreno et al. [56], which has been previously applied to Chilean populations [57,58]. This questionnaire, with high reliability (α = 0.89), measures the degree of enjoyment through 16 statements rated on a Likert scale from 1 (strongly disagree) to 5 (strongly agree).

#### 2.3.3. Cognitive Dimension

The level of knowledge and understanding of physical activity was measured using the cognitive dimension of the CAPL-2 instrument [54]. This tool assesses content related to PL (α = 0.903) and aligns with the Chilean Physical Education and Health curriculum [59]. It consists of five multiple-choice questions and one completion item, with results scored according to the guidelines established in the CAPL-2 manual [40,54].

#### 2.3.4. Cardiometabolic Risk Measures

Cardiometabolic risk variables were measured using standardized clinical procedures conducted under controlled conditions by trained nursing staff, including biochemical, anthropometric, and blood pressure assessments. Venous blood samples were collected by a private clinical laboratory in compliance with biosafety standards, following an overnight fast of 8 to 12 h. Anthropometric and blood pressure measurements were performed at educational institutions, in school infirmaries, or designated areas specifically prepared for this purpose.

Biochemical analyses included the determination of high-density lipoprotein cholesterol (HDL-C), triglyceride (TG), and fasting glucose (FG) levels. Blood pressure was assessed after a five-minute seated rest, with three consecutive measurements of systolic blood pressure (SBP) and diastolic blood pressure (DBP) taken at one-minute intervals using an automatic sphygmomanometer (Omron HEM-7130; Omron Healthcare, Inc., Bannockburn, IL, USA). For the statistical analysis, the mean of the last two readings was considered.

Anthropometric measurements included body weight, height, and waist circumference (WC). Body weight (kg) was measured using a digital scale with a precision of 0.1 kg (model 813, Seca GmbH & Co., Hamburg, Germany), with participants barefoot and wearing light clothing (shorts and a T-shirt). Height (cm) was assessed using a portable stadiometer accurate to 0.1 cm (model 213, Seca GmbH & Co., Hamburg, Germany). WC was measured using a flexible, self-locking measuring tape (Seca 201, Seca GmbH & Co., Hamburg, Germany), positioned at the level of the navel with a precision of 1 mm. Each measurement was performed twice, and a measurement was repeated if discrepancies were found. BMI was calculated from weight and height values using the formula weight/height^2^ [60,61].

Based on these variables, a continuous metabolic syndrome score (MetS-score) was constructed, as formulated by Gurka et al. [62] and previously applied to Chilean populations [63]. This indicator integrates the main components of metabolic syndrome (BMI, SBP, HDL, TG and FG), allowing continuous assessment of cardiometabolic risk. To identify participants with or without cardiometabolic risk, the Single Point Insulin Sensitivity Estimator (SPISE ≤ 5.4) was used, a tool validated in Chilean children and adolescents [64]. The SPISE value was calculated according to the formula described by Paulmichl et al. [65]:

$$\frac{600\times HDL^{0.185}}{TG^{0.2}\times BMI^{1.338}}$$

### 2.4. Co-Variable

#### Somatic Maturation

Peak height velocity (PHV) was evaluated using the equation described by Mirwald et al. [66]: PHV = −9.236 + (0.0002708 × (leg length × sitting height)) − (0.001663 × (age × leg length)) + (0.007216 × (age × sitting height)) + (0.02292 × (weight/height × 100)).

### 2.5. Intervention

Participants were randomly assigned, using simple randomization, to two experimental groups and one control group. Those allocated to the experimental groups were invited to participate in 48 supervised training sessions over 16 weeks, consisting of three sessions per week, each lasting approximately 60 min. Each session began with a 15 min standardized warm-up that included joint mobility and movement exercises performed with and without a ball. The main part of the session lasted approximately 40 min. Each session concluded with a 5 min cool-down consisting of low-intensity aerobic activities and stretching exercises. All sessions were monitored using Polar^®^ Verity Sense devices (Polar Electro Oy Inc, Kempele, Finland). The characteristics of each group were as follows:

#### 2.5.1. Small-Sided Soccer Games (SSG)

The SSG-based training protocol was designed based on a pilot study, in which participants were estimated to sustain vigorous intensity for approximately 80% of each session (>77% of maximum heart rate) [67,68]. The training design was structured according to previous evidence indicating that specific conditions enhance the effectiveness of this format. A fractional training model was implemented [58], consisting of 6–7 sets of 4 min. each, interspersed with 2 min rest intervals, on a reduced indoor playing area of 9 × 18 m. [30]. The number of players on the field varied between 2 vs. 2, 3 vs. 3, and 4 vs. 4 formats [69]. Game conditions were established to enhance play fluidity and intensity, such as individualized marking tasks [70], requiring full offensive participation for goal validation, and the use of multiple balls to minimize interruptions. Additionally, positive verbal encouragement was provided by the coach [71] to stimulate engagement and motivation during the game. The participants freely used their technical and tactical skills throughout the game.

#### 2.5.2. Traditional Soccer Group (TSG)

The training protocol for the TSG was based on Wang et al. [72] with minor modifications. The sessions consisted of traditional soccer training conducted on an indoor futsal surface (15 × 25 m.). The main 40 min portion of each session was structured and supervised by an instructor, focusing on the following technical skills: 10 min of dribbling, 10 min of passing, 10 min of dribbling again, followed by 10 min of standardized 5 vs. 5 gameplay according to the official rules. Exercise intensity was monitored during each session using a Polar^®^ Verity Sense device (Polar Electro Oy Inc.).

#### 2.5.3. Control Group (CG)

Participants assigned to the CG did not participate take part in any experimental intervention or weight reduction treatment (nutritional, pharmacological, or structured exercise). They were instructed to maintain their usual daily activities throughout the study period.

### 2.6. Procedures

Data collection was conducted over two weeks. During the first week, and after prior coordination, a mobile laboratory from a private clinic visited each school to collect blood samples for cardiometabolic risk assessments. Subsequently, a session was held in which participants completed self-report questionnaires and received an accelerometer, which they were required to wear according to the protocol for seven consecutive days. In the second week, anthropometric measurements were taken, and the accelerometers were retrieved. Finally, in a separate session, following the familiarization phase, physical fitness tests were administered to each participant in the following order: HGS, HJ, and 6MWT. Data collection was conducted by physical education teachers and the research team, following standardized protocols for each test.

### 2.7. Statistical Analysis

The sample size was calculated using G*Power v.3.1.9.6 and was based on the primary aim of the research project—to detect a significant treatment effect from baseline to four months post-intervention. Assuming an effect size (f) of 0.45 [33], a significance level of 5%, and statistical power of 80%, the analysis indicated that 17 participants per group were required (*n* = 51). Participant characteristics were described using descriptive statistics, expressed as mean and standard deviation (SD). All outcome variables were standardized by calculating the z-scores for the baseline values, subtracting the mean and dividing by the SD. Post-intervention z-scores were calculated relative to the baseline mean and SD as a standardized measure of the effect size [73,74]. Effect sizes were interpreted following Hopkins et al. [75]: trivial (<0.20), small (0.20–0.59), moderate (0.60–1.19), large (1.20–1.99), very large (2.00–3.99), and extremely large (≥4.00). Each outcome score was winsorized (when necessary) to minimize the influence of outliers [73,74]. Analysis of covariance (ANCOVA) models were constructed, including post-intervention outcome values as dependent variables (i.e., exercise vs. control), group as a fixed factor, and baseline levels of the outcome as a covariate [74]. All statistical analyses were performed using R (version 4.0.0; R Project for Statistical Computing). Statistical significance was set at *p* < 0.05.

## 3. Results

**Figure 1:**
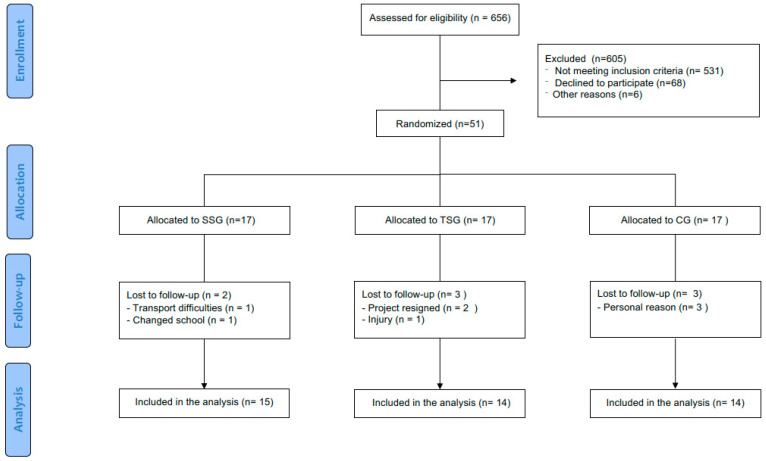
Flow diagram of participant enrollment, group allocation, follow-up, and final analysis. Note: SSG: Small-Sided Soccer Games; TSG: Traditional Soccer Group; CG: Control Group.

No statistically significant differences were observed between the groups at baseline in terms of anthropometry, body composition, cardiometabolic risk, or physical fitness. During the intervention period, eight participants withdrew from the study: two from the SSG group, three from the TSG, and three from the CG. The characteristics of the participants who withdrew did not differ from those who completed the final assessments, and therefore did not affect group homogeneity. Regarding session intensity, assessed through heart rate, the SSG interventions were more demanding than the TSG sessions (mean %HRmax: 81.1 ± 9.5% vs. 76.7 ± 10.5%; *p* < 0.001). Participants showed a 43% adherence rate to the intervention, with no significant differences between the SSG and TSG (42.5 ± 23.8% vs. 43.0 ± 21.9%; *p* = 0.96) across 48 programmed sessions (Figure 1).

Regarding sociodemographic, cardiometabolic risk, and PL characteristics, baseline values showed no significant differences among the CG, SSG, and TSG. Participants presented a BMI within the overweight–obesity range (28.61 ± 3.45 kg/m^2^) and a PHV indicative of mid-pubertal maturation (−1.04 ± 1.29). Cardiometabolic risk variables, including SBP and DBP, remained within age-appropriate normal ranges (SBP = 111.59 ± 11.59 mmHg; DBP = 66.98 ± 6.65 mmHg). FG levels reflected normoglycemia (FG: 89.3 ± 4.71 mg/dL). In terms of lipid profile, average triglyceride and HDL cholesterol levels indicated a mildly altered metabolic condition (TG = 109.37 ± 48.44 mg/dL; HDL = 46.14 ± 10.21 mg/dL). The results for the PL variables are presented in Table 1.

**Figure 2:**
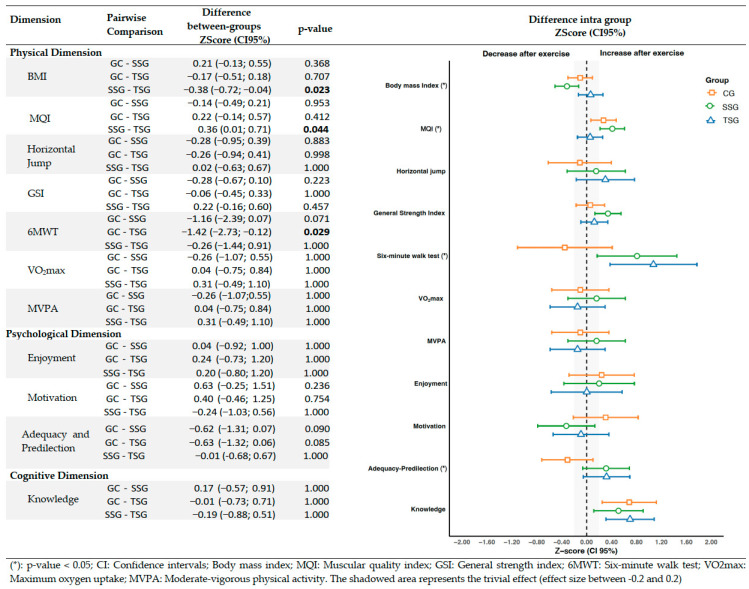
Effects of the exercise program on z-score changes between groups in physical literacy outcomes.

Figure 2 shows the differences in z-scores after the intervention (with their respective confidence intervals) among the three groups for each PL variable. The ANCOVA results revealed statistically significant differences among the SSG, TSG, and CG in BMI (F = 3.97(2); *p* = 0.03; η^2^p = 0.18), MQI (F = 3.30(2); *p* = 0.04; η^2^p = 0.08), 6MWT (F = 4.24(2); *p* = 0.02; η^2^p = 0.13), and Adequacy and Predilection (F = 3.40(2); *p* = 0.04; η^2^p = 0.15) (see Supplementary Materials). Similarly, the post hoc ANCOVA analysis indicated significant changes within the physical dimension, specifically in BMI and MQI for the SSG group compared to the TSG. A reduction in BMI was observed (−0.38 [−0.72; −0.04]; *p* = 0.023), along with an increase in muscle quality (MQI = 0.36 [0.01; 0.71]; *p* = 0.044). Additionally, significant differences in 6MWT were identified between the TSG and CG (−1.42 [−2.73; −0.12]; *p* = 0.029), indicating an improvement in functional performance in the TSG.

### 3.1. Intention-to-Treat Analysis

**Figure 3:**
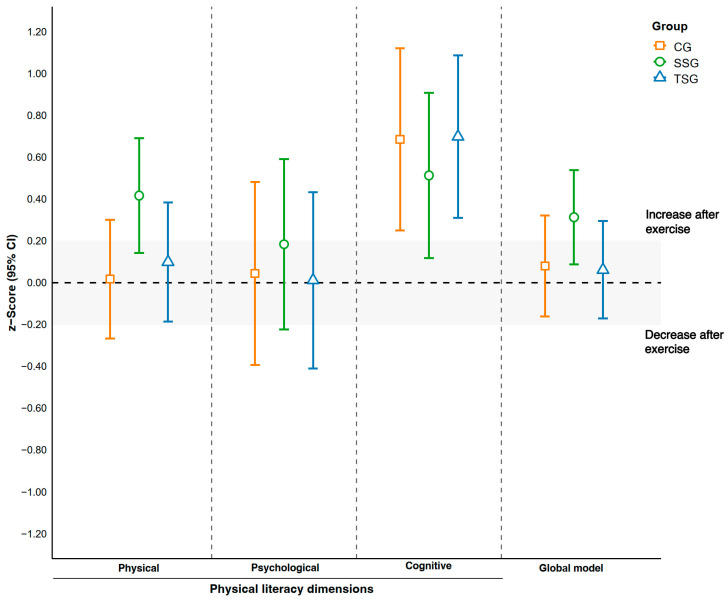
Effects of the exercise program on z-score changes across dimensions and the global model of physical literacy outcomes. Note. The shadowed area represents the trivial effect (effect size between −0.2 and 0.2).

Figure 3 presents the effect size results (z-scores) and their corresponding 95% confidence intervals (CI95%) for the PL dimensions. The ANCOVA analyses revealed no statistically significant differences among the groups in any dimension: physical (F = 1.56(2); *p* = 0.22; η^2^p = 0.08), psychological (F = 0.20(2); *p* = 0.82; η^2^p = 0.01), cognitive (F = 0.27(2); *p* = 0.77; η^2^p = 0.09), or in the overall PL model (F = 1.53(2); *p* = 0.23; η^2^p = 0.07). Although no intergroup differences reached statistical significance, within-group analyses showed a positive trend in the SSG group, with higher effects in the overall PL model (0.31 [0.08; 0.53]) compared to TSG (0.06 [−0.17; 0.29]) and CG (0.08 [−0.16; 0.32]). In the physical dimension, SSG also showed moderate improvements (0.41 [0.14; 0.69]) compared with TSG (0.09 [−0.18; 0.38]) and CG (0.01 [−0.26; 0.30]). For the psychological dimension, a small favorable effect was observed for SSG (0.18 [−0.22; 0.59]), while in the cognitive dimension, the largest effects were recorded in TSG (0.78 [0.37; 1.2]) and CG (0.72 [0.30; 1.15]), with lower values for SSG (0.36 [−0.05; 0.77]).

### 3.2. Per-Protocol Analysis

**Figure 4:**
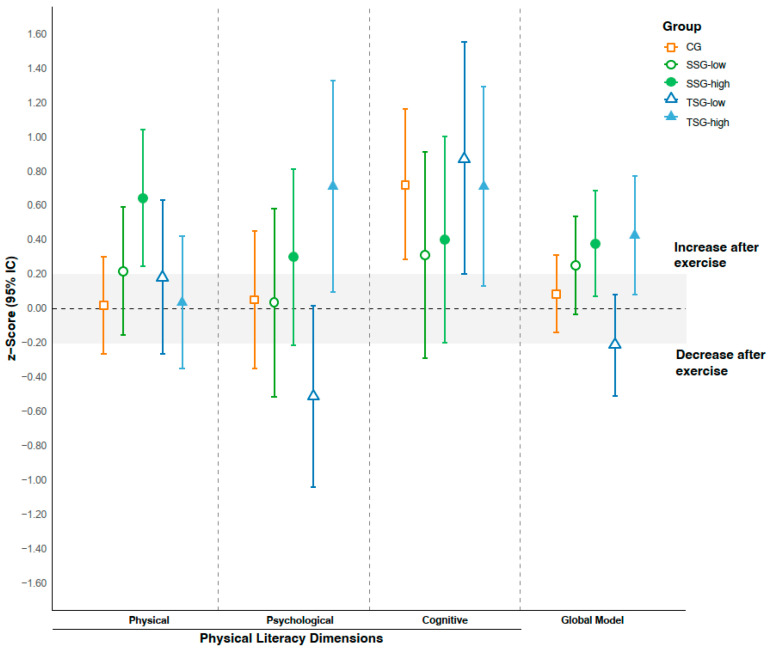
Effects of the exercise program on z-score changes across dimensions and the global model of physical literacy outcomes. Per-protocol analysis. Note. The shadowed area represents the trivial effect (effect size between −0.2 and 0.2).

Figure 4 shows the results of the per-protocol analysis. Subgroups for SSG and TSG were established based on program adherence. The terms SSG-low and TSG-low were used for participants who attended less than 50% of the sessions, while SSG-high and TSG-high referred to those with more than 50% attendance. A total of 47% (*n* = 7) of participants were classified as SSG-low and 43% (*n* = 6) as TSG-low. The within-group ANCOVA analysis revealed significant differences in the overall PL model (F = 2.91(4); *p* = 0.035; η^2^p = 0.26). The post hoc analysis indicated a non-significant trend between high- and low-adherence subgroups (SSG-high and TSG-high vs. TSG-low; *p* = 0.07 and *p* = 0.08, respectively). In the physical (F = 1.88(4); *p* = 0.13; η^2^p = 0.17) and psychological dimensions (F = 2.43(4); *p* = 0.06; η^2^p = 0.25), similar trends were observed, with a marginal difference between TSG-low and TSG-high (*p* = 0.05). The cognitive dimension showed no significant effects (F = 0.65(4); *p* = 0.63; η^2^p = 0.09). Regarding within-group effects in the overall PL model, the highest values were observed in the high-adherence subgroups: SSG-high (0.38 [0.07; 0.68]) and TSG-high (0.43 [0.08; 0.77]). In the physical dimension, the SSG-high group achieved the greatest effect (0.64 [0.24; 1.04]); in the psychological dimension, TSG-high reached the highest score (0.71 [0.13; 1.29]); and in the cognitive dimension, the highest value corresponded to TSG-low (0.87 [0.20; 1.55]).

## 4. Discussion

The aim of this study was to analyze whether recreational soccer, through SSG and TSG modalities, could promote improvements across the different dimensions of PL in adolescents with high cardiometabolic risk. The results of the study rejected the initial hypothesis; however, the findings showed that the overall PL model exhibited a small, statistically non-significant effect for the SSG group (ES = 0.31) compared with TSG and CG, which reported trivial effects (ES = 0.06 and 0.08, respectively). The effects observed within the PL model dimensions indicated that SSG performed better in two out of the three dimensions, showing a small effect for the physical dimension (ES = 0.41) and a trivial effect for the psychological dimension (ES = 0.18). In this context, the per-protocol analysis adjusted for adherence showed that the overall model presented a small effect in both TSG-high and SSG-high (ES = 0.43 and 0.38, respectively) and revealed significant within-group differences (F = 2.91(4); *p* = 0.035; η^2^p = 0.26). However, post hoc results only reached trend-level significance for the SSG-high and TSG-high groups compared with the TSG-low group (*p* = 0.07 and *p* = 0.08, respectively). The physical dimension showed a moderate effect in the SSG-high group (ES = 0.64), as did the psychological dimension in the TSG-high group (ES = 0.71), while the cognitive dimension for the TSG-low group (ES = 0.87).

The analysis of the overall model demonstrates that an intervention based solely on recreational soccer is insufficient to comprehensively enhance the multidimensional construct of PL within a three-dimensional theoretical framework. In contrast to our study, interventions that have incorporated multidimensional elements have shown significant effects across various dimensions of PL [76,77]. However, the evidence remains limited regarding the overall assessment of PL. In their meta-analysis, Carl et al. [33] identified that only 21.7% of the interventions evaluated multidimensional effects, and only three reported significant results. Furthermore, while Nezondet et al. [76] observed a 16% improvement in overall PL, Romero-Martínez et al. [77], despite finding changes in specific dimensions, did not report an effect on the global PL score. In line with this, our findings indicate that improvements in the physical dimension—specifically in BMI, MQI, and 6MWT—do not necessarily translate into increases in overall PL, reinforcing the complexity of the construct and the challenge of capturing its multidimensional nature [33].

On the other hand, the per-protocol analysis results for the overall PL model indicated that adherence is a potential determinant of program effectiveness. The findings revealed trends and some significant differences among participants with higher adherence (SSG-high and TSG-high) compared with those who participated less (<50%), which had not been observed in the intention-to-treat analyses. This reinforces the importance of high adherence to facilitate adaptation processes and improvements in the evaluated parameters. In this context, poor reporting of dropout rates [78,79] can bias interpretations of intervention efficacy, effectiveness and transferability [9]. While studies such as Telford et al. [80] and Nezondet et al. [76] did not report adherence despite implementing long-term interventions, others require ≥ 50% attendance [22,81], a criterion also applied here.

The low participation observed in the program may be attributable to a combination of behavioral and contextual barriers. Behaviorally, adolescents’ tendency to prioritize immediate rewards [82] hinders the sustained commitment required for PL. From the perspective of Self-Determination Theory [83], which suggests the theoretical model of PL [14], behavior change is driven by autonomous motivation and enjoyment, which emerge when basic psychological needs are satisfied, providing deeper and more sustainable internal rewards than impulsive gratification. However, in adolescents with elevated BMI, intrinsic motivation is typically lower [84], which may have negatively influenced adherence and the effectiveness of the intervention. This tendency may help explain why obesity management programs often show limited effectiveness in this developmental stage [85,86]. Contextually, the intervention was implemented in late 2023, immediately after school hours, coinciding with residual post-COVID-19 disruptions to healthy behaviors and the reestablishment of school routines [87]. Regionally, a 39% absenteeism rate [88] reflected these difficulties. Although attendance data were not collected, such contextual factors likely hindered adherence and consistent participation in the intervention.

### 4.1. Physical Dimension

The SSG effect on the physical dimension did not show significant between-group differences; however, it demonstrated a small effect (0.33 [0.04; 0.61]) compared with TSG and CG (0.09 [−0.18; 0.38] and 0.01 [−0.26; 0.30], respectively), which is consistent with existing evidence from interventions using SSG as a strategy to improve physical components [23]. When comparing groups within the physical dimension variables, significant differences were observed in BMI and muscle quality, specifically between SSG and TSG (ES: −0.38, *p* = 0.023; ES: 0.36, *p* = 0.044, respectively). The latter is particularly relevant in populations with abdominal obesity, as lower muscle efficiency and greater intramuscular fat infiltration increase metabolic dysfunction and inflammation [89,90], altering myokine secretion and exacerbating insulin resistance and cardiometabolic risk [91,92]. Regarding BMI, the results were similar to those reported by Nezondet et al. [76] for post-intervention z-score changes (−0.3; *p* = 0.003). Although the SSG strategy has demonstrated effectiveness in reducing BMI [79], it is important to note that the characteristics of the sample with high cardiometabolic risk interact with the individual in a negative or inverse spiral [14,93], hindering the development of the physical dimension, motivation, enjoyment, and adherence. Meanwhile, the TSG showed significant differences in cardiorespiratory fitness, as measured by the 6MWT, compared with the CG (ES: −1.42; *p* = 0.029), whereas no significant differences were found between TSG and SSG.

Although the other variables of the physical dimension (GSI, SH, VO_2_max, and MVPA) did not show significant changes, it is noteworthy that the MVPA data collected are similar to the intervention effects reported by Telford et al. [80] and Nezondet et al. [76] regarding MVPA time modifications in PL-based interventions (*p* = 0.058 and *p* = 0.36, respectively), in normal-weight children (*n* = 318 over 33 weeks) and adolescents with abdominal obesity (*n* = 11 over 9 months), respectively. In this regard, the non-significant effect may be attributed both to the characteristics of the sample and to the high variability observed, as well as to the limitations of wrist-worn accelerometers which, although practical and well tolerated by participants, may substantially underestimate soccer-specific movements—particularly those involving intermittent bursts of intensity or minimal arm displacement [47,94]—in addition to the inherent limitations of accelerometer-based MVPA measurements [95]. Nevertheless, maintaining MVPA levels after the intervention is relevant, as it suggests that a recreational soccer-based program, in addition to enhancing physical literacy, may prevent the typical decline in MVPA during adolescence [96], a finding supported by previous studies in adolescent and university populations [97,98].

### 4.2. Psychological and Cognitive Dimensions

The psychological dimension did not show significant changes between groups (0.18 [−0.22; 0.59]); however, the variable “adequacy and predilection” toward physical activity showed significant differences (F = 3.40(2); *p* = 0.04), with a small effect in favor of SSG and TSG (0.31 [−0.05; 0.68] and 0.32 [−0.05; 0.69], respectively) compared with CG (−0.30 [−0.71; 0.10]). These results are consistent with those reported by Liu and Chen [99], who found a small effect (d = 0.26) for the same variable after eight weeks of intervention. Regarding motivation and enjoyment, our findings are consistent with those of Muñoz-Urtubia et al. [84] in children with elevated BMI, showing an inverse relationship between BMI and motivation (ES = 0.198), which in turn represents a direct barrier to motivation and enjoyment, and indirectly to program adherence. Although recreational soccer is usually a highly motivating activity for adolescent boys [84], in this study, it did not produce a significant increase in motivation (*p* = 0.21) in any of the groups. This result can be understood in light of adolescents’ tendency to prioritize immediate rewards over long-term benefits [82], a behavioral pattern that acts as a barrier to the promotion of PL and to the behavior change processes proposed by Self-Determination Theory [83]. This is particularly relevant considering that intrinsic motivation is a core component of the physical literacy framework proposed by Cairney et al. [14].

Finally, the cognitive dimension did not show statistically significant effects between the experimental groups; however, moderate effects were observed in SSG (0.51 [0.12; 0.91]), TSG (0.70 [0.31; 1.09]), and CG (0.69 [0.25; 1.12]). Similarly to the pilot study by Liu and Chen [99], in which an 8-week intervention led to small but positive changes in the cognitive dimension (g = 0.15), our results also demonstrated moderate effect sizes. It is important to note that Liu and Chen [99] included targeted sessions addressing each dimension of physical literacy as part of their intervention, thereby explicitly incorporating the cognitive component into the program design. In contrast, our study evaluated the effects of a recreational soccer program without implementing a specific cognitive intervention. Thus, the observed improvement in the cognitive dimension may be more closely related to participants’ increased familiarity with curricular content corresponding to their educational level rather than to the direct effects of the intervention itself.

### 4.3. Practical Considerations

The multidimensional nature of PL [14] presents a significant challenge for designing interventions that comprehensively assess an individual’s level of PL, as reflected in other assessment frameworks [40,100,101]. Our approach aligns with multidimensional assessment models that emphasize the predominance of the physical dimension over the cognitive and psychosocial dimensions. Although there is consistent evidence supporting SSG as an integrated strategy for fostering the transfer of knowledge and the development of motor, affective, and social competencies within the PL framework, our findings underscore the need to continue developing multidimensional intervention strategies that can enhance adherence among clinical populations such as adolescents at high cardiometabolic risk.

### 4.4. Limitations and Recommendations

This study has several limitations that should be considered when interpreting the results. (i) The adherence rate to the intervention program was low (43%), reflecting the persistent challenge of maintaining sustained participation in real-world settings, particularly among adolescents with high cardiometabolic risk. This low adherence may have limited both the magnitude and depth of the observed effects. (ii) The sample size was small and comprised exclusively of male adolescents, which restricts the generalizability of the findings to female or mixed populations. Future studies should increase the sample size and include representative samples of both sexes to enhance the applicability of the PL model in diverse and real-world contexts. (iii) Future research should also strengthen the assessment of the cognitive dimension and incorporate the evaluation of a social dimension, as well as measures of actual and perceived motor competence (iv) The use of wrist-worn accelerometers, although practical and well-tolerated by participants, may have led to an underestimation of actual physical activity levels, particularly for activities involving limited arm movement or intermittent intensity typical of soccer play.

It is recommended that future research designs interventions that not only include structured playful strategies but also integrate intentional and differentiated pedagogical approaches to stimulate the dimensions of physical literacy (PL) in a balanced manner. Furthermore, considering the role of the educator or coach, as well as the pedagogical models they apply, may be crucial for enhancing participants’ autonomous motivation, motivational climate, and affective engagement. In addition, future studies should explicitly analyze the relationship between PL levels and clinical indicators of cardiometabolic risk using statistical models that allow the establishment of causal links. This would help to strengthen the multidimensional approach to PL as a preventive tool in clinical populations, particularly among adolescents at high cardiometabolic risk. This recommendation is especially relevant for populations with clinical conditions or special needs, in which motor trajectories may be influenced by multiple individual and contextual factors. Based on the experience of our intervention, it is also recommended that future studies involving adolescents with high cardiometabolic risk include intermediate measurement points to monitor program effects over time and to more consistently track adherence processes in this population.

## 5. Conclusions

Recreational soccer, whether implemented in SSG or TSG formats, appears insufficient on its own to produce meaningful improvements in physical literacy among adolescents with high cardiometabolic risk. Nevertheless, participants who demonstrated higher adherence tended to achieve better outcomes in physical literacy, particularly in the physical and psychological domains, where SSG yielded more consistent positive effects. The modest magnitude of these improvements, together with the generally low adherence observed, highlights the need to refine both the design and implementation of such interventions. Future studies should adopt a multidimensional and pedagogically intentional framework that integrates strategies actively engaging all components of physical literacy—physical, cognitive, and affective—while fostering adherence to enhance the effectiveness and long-term sustainability of outcomes in adolescents at high cardiometabolic risk.

## Figures and Tables

**Table 1:** Participant characteristics by intervention group.

	All (*N* = 43)	CG (*n* = 14)	SSG (*n* = 15)	TSG (*n* = 14)	
	Mean (SD)	Mean (SD)	Mean (SD)	Mean (SD)	*p*-Value
Sociodemographic variables					
Age (year)	13.16 (1.40)	13.07 (1.47)	12.97 (1.31)	13.44 (1.45)	0.605
Weight (kg)	76.41 (15.36)	75.15 (14.46)	78.57 (14.6)	75.52 (17.52)	0.768
Height (cm)	162.62 (10.04)	162.04 (8.68)	163.54 (11.20)	162.28 (10.63)	0.906
PHV	−1.04 (1.29)	−1.09 (1.19)	−1.04 (1.27)	−0.99 (1.48)	0.979
BMI (kg/m^2^)	28.61 (3.45)	28.41 (3.48)	29.1 (2.55)	28.30 (4.25)	0.722
Cardiometabolic variables					
SBP (mmHg)	111.59 (11.59)	108.82 (9.48)	113.82 (13.75)	112.12 (11.27)	0.428
DBP (mmHg)	66.98 (6.65)	65.97 (6.08)	66.44 (7.07)	68.53 (6.88)	0.506
FG (mg/dL)	89.3 (4.71)	89.49 (5.11)	90.46 (4.52)	87.98 (4.47)	0.290
HDL-C (mg/dL)	46.14 (10.21)	48.2 (12.37)	44.29 (8.04)	46.18 (10.31)	0.572
TG (mg/dL)	109.37 (48.44)	98.73 (41.48)	106.94 (41.56)	121.18 (59.55)	0.475
SPISE (a.u)	5.57 (1.22)	5.66 (1.28)	5.37 (0.88)	5.68 (1.49)	0.664
Physical dimensión					
MQI (a.u)	2.14 (0.65)	2.12 (0.54)	2.11 (0.62)	2.19 (0.79)	0.944
Horizontal jump (cm)	130.60 (24.84)	131.43 (23.84)	132.41 (23.02)	127.94 (28.69)	0.885
GSI (a.u)	0.03 (0.92)	0.10 (0.93)	0.00 (0.93)	0.01 (0.96)	0.951
6MWT (m)	529.67 (123.5)	566.33 (50.57)	555.91 (56.75)	546.72 (42.85)	0.490
VO2max (mL/kg/min)	24.05 (6.22)	25.88 (4.41)	24.75 (3.9)	24.79 (4.81)	0.698
MVPA/DAY (min)	14.8 (11.23)	9.87 (9.82)	17.11 (12.67)	16.11 (10.23)	0.179
Psychological dimension					
Enjoyment (a.u)	65.29 (10.87)	67.14 (7.63)	64.38 (7.07)	64.53 (16.08)	0.589
Adequacy and Predilection (a.u)	12.27 (2.8)	12.78 (2.25)	11.19 (3.4)	12.84 (2.44)	0.279
Motivation (a.u)	11.41 (2.31)	10.75 (2.34)	12.13 (1.88)	11.31 (2.59)	0.228
Cognitive dimension					
Knowledge (a.u)	5.89 (2.18)	6 (2.32)	5.13 (2.03)	6.5 (2.1)	0.203

*p*-value < 0.05; SD: Standard deviation; PHV: Peak high velocity; BMI: Body mass index; DBP: Diastolic blood pressure; SBP: Systolic blood pressure; FG: fasting glucose; HDL-C: Lipoprotein cholesterol; TG: Triglyceride; SPISE: Single point insulin sensitivity estimator; GSI: General Strength index; 6MWT: Six-minute walk test; VO2max: Maximum oxygen uptake; MVPA: Moderate-vigorous physical activity; MQI: muscular quality index; bpm: Beats per minute; mmHg: Milligrams of mercury; µU/mL: Micro-units per milliliter; mg/dL: Milligrams per deciliter; cm: Centimeter; m: Meters; mL/kg/min: Milliliters per kilogram per minute; a.u: Arbitrary units.

## Data Availability

The data are not publicly available due to privacy or ethical restrictions.

## Supplementary Materials

The following supporting information can be downloaded at: https://www.mdpi.com/article/10.3390/sports13120423/s1, Table S1: Analysis of between-group differences in z-score data.
