# Supplementary material for: Can Recreational Soccer Improve Physical Literacy Dimensions Among Adolescents with High Cardiometabolic Risk?
Source: Sports (Basel). 2025 Dec 2;13(12):423. doi: 10.3390/sports13120423 (PMC12737039; doi:10.3390/sports13120423)
Supplement: Supplementary file 1 [file sports-13-00423-s001.zip › Suplem 1.pdf]

Sumpl.1

Table S1. Analysis of between-group differences in z-score data

Dimension	GC Z-score (95%CI)	SSG Z-score (95%CI)	TSG Z-score (95%CI)	F(gl); p-valor; η²p
**Physical Dimension**				
BMI	-0.10 (-0.30; 0.10)	-0.31 (-0.50; -0.12)	0.07 (-0.13; 0.26)	**3.97(2); 0.03; 0.18**
MQI	0.27 (0.07; 0.48)	0.41 (0.22; 0.61)	0.06 (-0.15; 0.26)	**3.30(2); 0.04; 0.08**
Horizontal Jump	-0.08 (-0.47; 0.32)	0.21 (-0.16; 0.57)	0.19 (-0.19; 0.56)	0.69(2); 0.51; 0.01
GSI	0.06 (-0.17; 0.29)	0.34 (0.13; 0.56)	0.12 (-0.09; 0.34)	1.91(2); 0.16; 0.01
6MWT	-0.35 (-1.11; 0.41)	0.81 (0.17; 1.45)	1.07 (0.37; 1.77)	**4.24(2); 0.02; 0.13**
VO_2max	-0.10 (-0.56; 0.36)	0.16 (-0.30; 0.62)	-0.14 (-0.59; 0.30)	0.54(2); 0.59; 0.04
MVPA	-0.10 (-0.56; 0.36)	0.16 (-0.30; 0.62)	-0.14 (-0.59; 0.30)	0.54(2); 0.59; 0.04
**Psychological Dimension**				
Enjoyment	0.24 (-0.28; 0.77)	0.20 (-0.36; 0.77)	0.00 (-0.57; 0.57)	0.22(2); 0.81; 0.01
Adequacy and Predilection	-0.31 (-0.72; 0.11)	0.31 (-0.06; 0.69)	0.32 (-0.05; 0.69)	**3.40(2); 0.04; 0.15**
Motivation	0.31 (-0.21; 0.83)	-0.32 (-0.78; 0.13)	-0.09 (-0.54; 0.36)	1.65(2); 0.21; 0.00
**Cognitive Dimension**				
Knowledge	0.69 (0.25; 1.12)	0.51 (0.12; 0.91)	0.70 (0.31; 1.09)	0.27(2); 0.77; 0.09

p-valor < 0.05; BMI: Body mass index; MQI: Muscular Quality index; GSI: General Strength index; 6MWT: Six-minute walk test; VO_2max: Maximum volume of oxygen; MVPA: Moderate-vigorous physical activity
